# APPLICATION OF A TWO-COAT SELF-ASSEMBLING PEPTIDE HEMOSTATIC GEL TECHNIQUE IN MANAGING ENDOSCOPIC SPHINCTEROTOMY-RELATED BLEEDING: A CASE REPORT

**DOI:** 10.1590/S0004-2803.24612024-133

**Published:** 2025-04-04

**Authors:** Koichi SOGA, Ikuhiro KOBORI, Masaya TAMANO

**Affiliations:** * Dokkyo Medical University Saitama Medical Center, Department of Gastroenterology, Minami-Koshigaya, Koshigaya, Saitama, Japan.

## Case presentation

Endoscopic sphincterotomy (EST)-related bleeding is a serious adverse event with an incidence of 0-27%^1^. A novel self-assembling peptide matrix-forming gel (SAP-gel; Purestat, 3-D Matrix Ltd., Tokyo, Japan) has been reported to be easy to apply, simple, safe, and effective for achieving hemostasis in gastrointestinal bleeding^2^. SAP-gel was applied at the bleeding point using a delivery catheter inserted through the endoscope accessory channel. SAP-gel forms a collagen-like fibrous network upon exposure to fluids under physiological conditions. This network functions as a mechanical barrier, occludes bleeding points, and promotes tissue regeneration^2^. SAP-gel applied as a hemostatic treatment for intraoperative bleeding occurring during EST can also prevent postoperative bleeding^3^.

**FIGURE 1:**
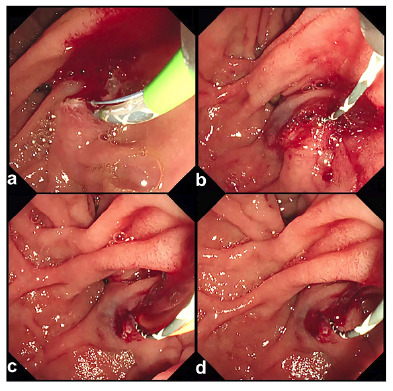
Endoscopic sphincterotomy (EST)-related bleeding associated with endoscopic retrograde cholangiopancreatography (ERCP) procedures for this case.

**FIGURE 2:**
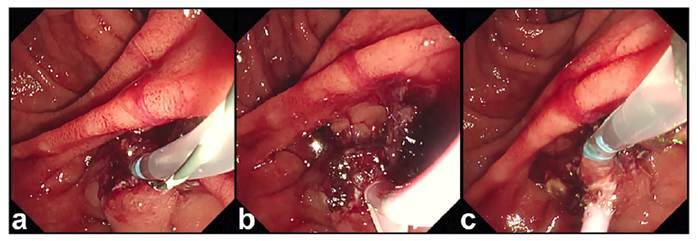
Application of two coats of a novel self-assembling peptide matrix-forming gel (SAP-gel) to achieve hemostasis.

A 58-year-old Japanese woman underwent endoscopic retrograde cholangiopancreatography (ERCP) to investigate irregular bile duct stenosis. Following EST, the patient experienced papillary bleeding. Initial hemostasis was achieved using pressure hemostasis with an extraction balloon and cooling of the duodenal lumen with cold water. However, re-bleeding occurred when the biopsy forceps were inserted into the bile duct (Figure 1). Two coats of SAP-gel were used to prevent re-bleeding. First, 1 mL of SAP-gel was applied to the papilla to ensure deep penetration before stent placement, allowing the gel to reach deeper than the papilla. An additional 2 mL was applied after stent placement to enhance hemostasis around the papilla (Figure 2). Recurrent EST-related bleeding was not observed (E-VIDEO).

A 58-year-old Japanese woman underwent therapeutic endoscopic retrograde cholangiopancreatography (ERCP) to investigate an enlarged pancreas and irregular bile duct stenosis. After EST, duodenal papillary bleeding occurred (**A**). Hemostasis was initially achieved by applying pressure using an extraction balloon. The duodenal lumen was then cooled with cold water. Primary hemostasis was confirmed using this procedure (**B**). Re-bleeding was induced using biopsy forceps (**C**), and temporary hemostasis was achieved using the same initial procedure (**D**).

First, 1 mL SAP-gel was applied to the papilla before the plastic stent was placed to ensure deeper application (**A**). The remaining 2 mL SAP-gel will be applied after the plastic stent placement (Suzaku, Kaneka, Tokyo, Japan) (**B**). Recurrent EST-related bleeding was not observed after the procedure (**C**).

SAP-gel can achieve hemostasis by rapidly forming nanofibers into a peptide gel upon contact with blood. SAP-gel potentially reduces inflammatory cytokines while promoting the expression of factors associated with wound healing^4^. The required gel volume varied depending on the intervention. On average, 2.4 mL is needed for hemostasis and 2.7 mL for delayed blee­ding prevention^2^. For EST-related bleeding, less SAP gel is required. Since 3 mL often exceeds the need at the duodenal papillae, efficient resource use is crucial. The two-coating method of SAP-gel potentially reduces the risk of re-bleeding compared to traditional methods such as clipping or coagulation. Additionally, it offers advantages in terms of cost-effectiveness and ease of application.

We consider this procedure as a prospective case report because the two-coating method of SAP-gel is expected to be useful for utilizing limited medical resources, to have a synergistic effect on preventing re-bleeding, and to promote tissue healing within deeper bile ducts that are not directly accessible by the endoscope. Given the limitations of this report to a single case, further validation through a more extensive case series is warranted. Further prospective case studies are needed to confirm the usefulness of this method. 
